# “Plugged” Anterior Inferior Cerebellar Artery Aneurysm Causing Facial Palsy, Hearing Loss, and Subarachnoid Hemorrhage Treated by a Translabyrinthine Approach

**DOI:** 10.7759/cureus.12282

**Published:** 2020-12-25

**Authors:** Buqing Liang, Thomas Brammeier, Jason Huang, Ethan A Benardete

**Affiliations:** 1 Neurosurgery, Baylor Scott & White Medical Center - Temple, Temple, USA; 2 Otolaryngology, Baylor Scott & White Medical Center - Temple, Temple, USA

**Keywords:** aneurysmal subarachnoid hemorrhage, translabyrinthine, aneurysm, aica

## Abstract

Anterior inferior cerebellar artery (AICA) aneurysms are rare, less than 1%-2% of all intracranial aneurysms. Aneurysms of the distal AICA are even less common and can present with hearing loss and facial paralysis because of their relationship with the internal auditory canal (IAC). A 65-year-old male was followed for fluctuating left facial weakness and left-sided hearing loss for over a year. Serial magnetic resonance imaging (MRI) scans showed a mass near the left IAC, thought to be a vestibular schwannoma. Just prior to his next clinic visit, the patient deteriorated suddenly from a subarachnoid hemorrhage. Cerebral angiography revealed a 5.5 mm saccular aneurysm at the distal left AICA, which was clip ligated via a translabyrinthine (TL) approach. The patient had a good functional outcome (modified Rankin Scale [mRS] 1) after 30 days despite persistent left facial weakness. Stable obliteration of the aneurysm was demonstrated by cerebral angiography postoperatively. Distal AICA aneurysms are rare and can have a similar presentation to tumors in the cerebellar pontine angle. Because of the unique anatomy of the distal AICA, open clip ligation via a TL approach is an effective method to secure these aneurysms.

## Introduction

Anterior inferior cerebellar artery (AICA) aneurysms are rare and comprise less than 1%-2% of all intracranial aneurysms [1-6]. AICA aneurysms that are distal to basilar artery (BA)-AICA junction are even less common [7-11]. We describe a patient who presented with acute facial weakness and hearing loss and was found to have a mass in the IAC, thought to be a small vestibular schwannoma (VS), a common cause of hearing loss. Much later, when the patient suffered a subarachnoid hemorrhage (SAH), it became clear that the lesion was a distal AICA aneurysm. The patient underwent successful clipping of the aneurysm via a translabyrinthine (TL) approach. In this case report, we discuss the diagnosis and management of distal AICA aneurysms and the benefits of the TL approach.

## Case presentation

**Figure 1:**
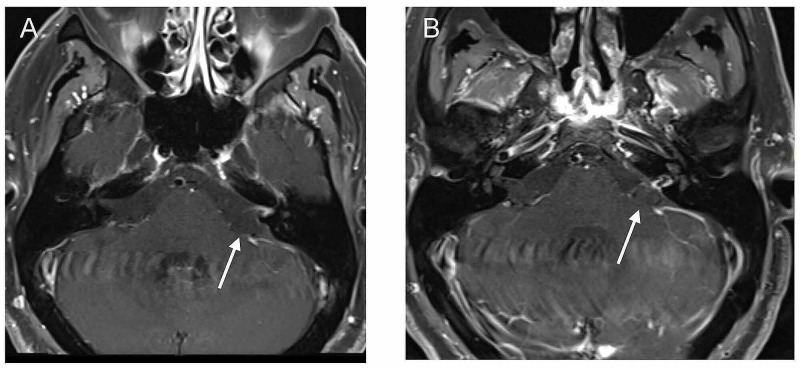
Pre-rupture MRI MRI of the brain with contrast revealed a contrast-enhancing mass at the left IAC (A), which slightly enlarged six days prior to the SAH with central non-enhancement (B). MRI, Magnetic resonance imaging; IAC, internal auditory canal; SAH, subarachnoid hemorrhage.

A 65-year-old male with a past medical history of hypertension and hyperlipidemia presented to the emergency department with a sudden onset of left ear pain, tinnitus, decreased hearing, and facial paralysis after heavy-lifting. A computed topography (CT) scan of the brain was normal. The patient was given a tentative diagnosis of Ramsay Hunt syndrome and discharged home with prednisone and valacyclovir. Magnetic resonance imaging (MRI) of the brain on the following day revealed a 7 mm x 3.8 mm x 3 mm enhancing mass near the left IAC, interpreted as a VS. The patient was referred to the otolaryngology service and followed with serial MRIs of the brain at four-month, eight-month, and 18-month intervals. The last MRI showed an increase of the size to 8 mm x 4 mm x 4 mm with central non-enhancement (Figure 1). At his last clinic visit, he had House-Brackmann III left facial weakness. An audiogram revealed no response to either tones or speech recognition on the left.

**Figure 2:**
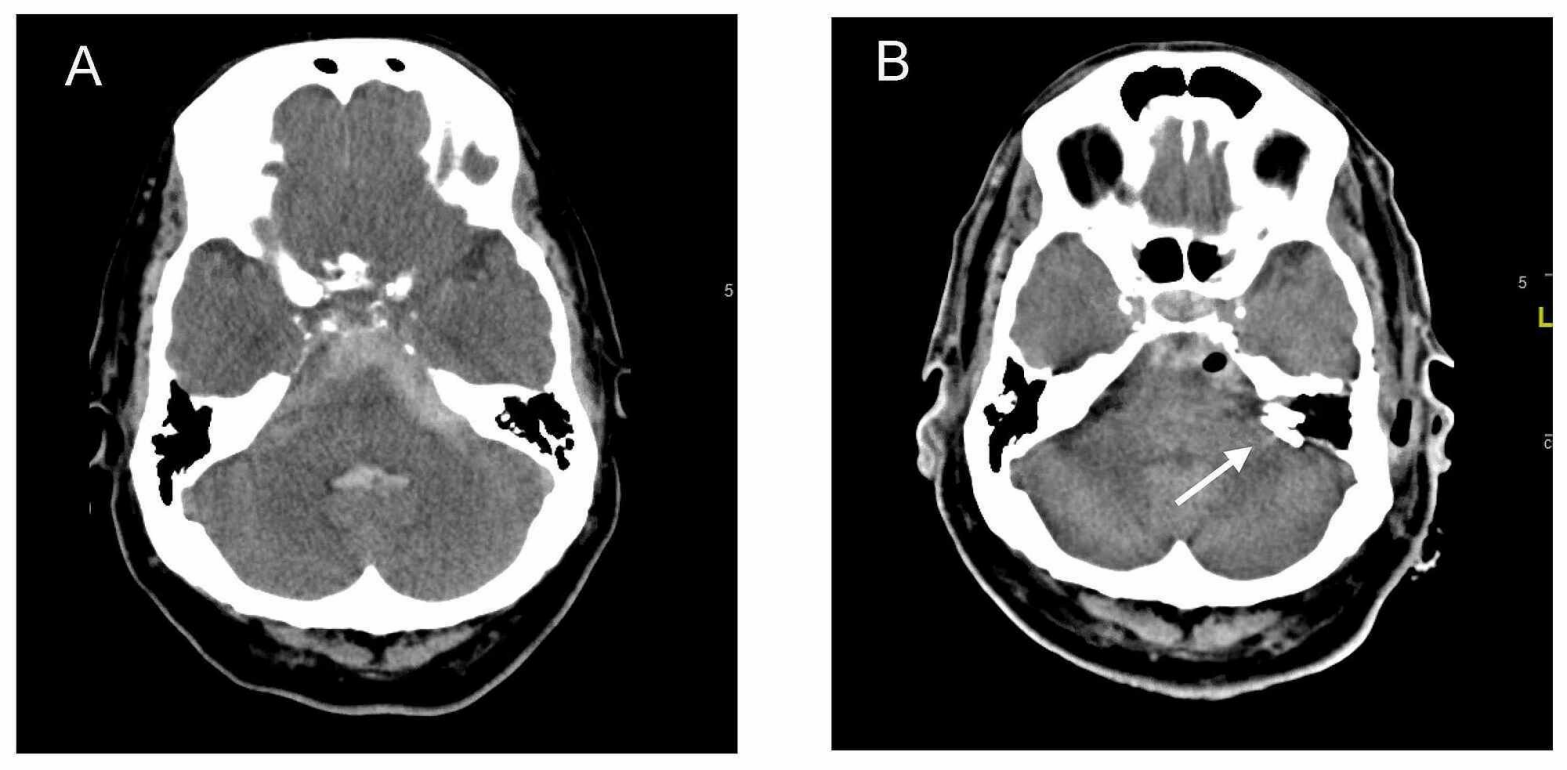
Pre- and post-op CT head Admission CT head without contrast revealed a Fisher grade 4 SAH with extension of hemorrhage into the fourth ventricle. The SAH pattern is more localized at the left cerebellopontine angle cistern, close to the internal auditory canal (A). Post-operative CT head revealed the location of the clips near the left IAC (B). CT, Computed topography; IAC, internal auditory canal; SAH, subarachnoid hemorrhage.

**Figure 3:**
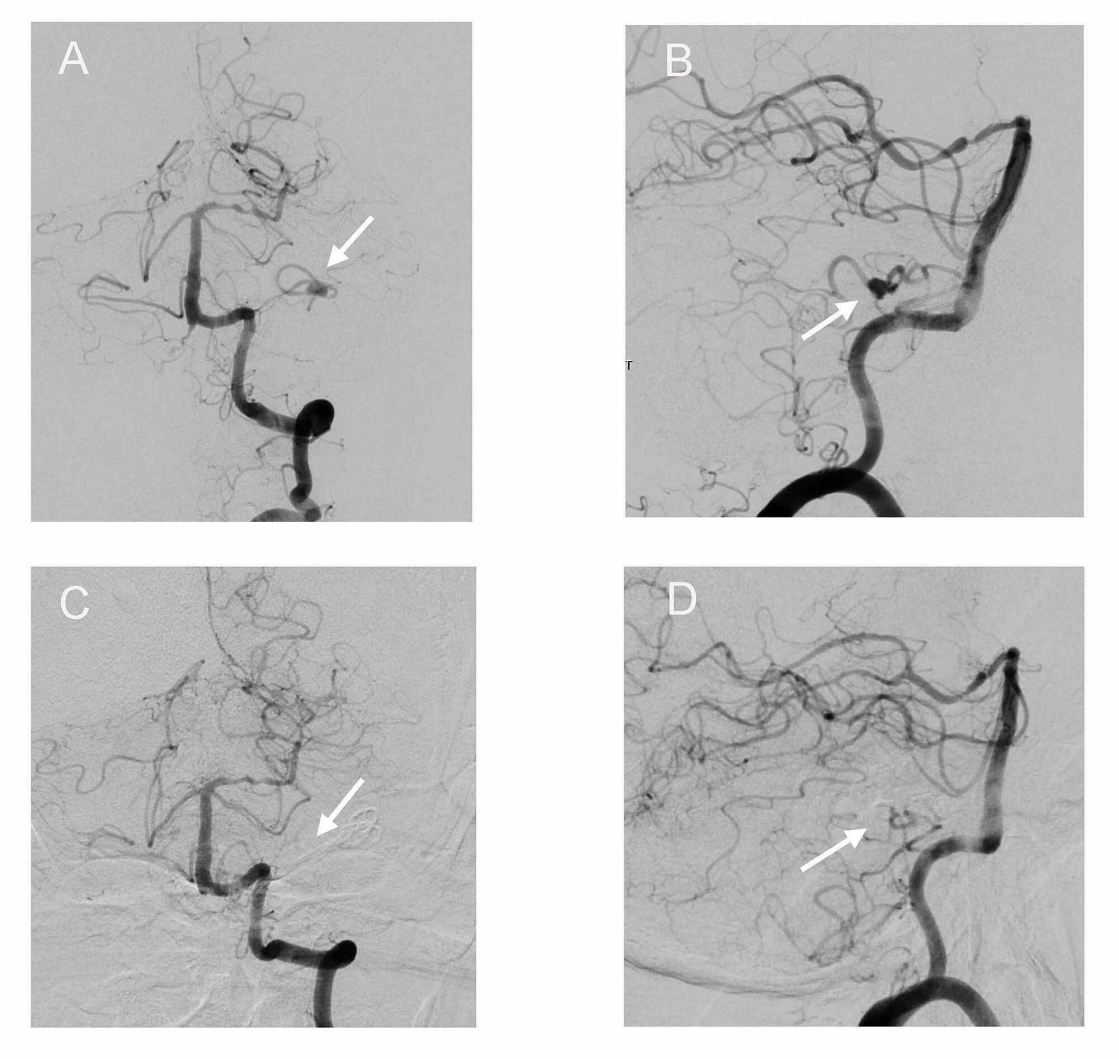
Pre- and post-op DSAs Left vertebral artery DSA revealed a distal AICA aneurysm close to the A2 meatal loop AP (A) and lateral (B). Left vertebral artery injection revealed no residual aneurysm and good AICA territory filling at two weeks post-clipping AP (C) and lateral (D). DSA, Digital subtraction angiography; AICA, anterior inferior cerebellar artery.

One week before his next scheduled clinic follow-up, the patient was found unconscious at home (Hunt and Hess Grade 4). A non-contrast CT scan revealed a Fisher Grade 4 SAH (Figure 2). He was intubated for airway protection, transferred to the hospital, and admitted to the neuro-ICU. An external ventricular drain was placed, and digital subtraction angiography (DSA) revealed a saccular left distal AICA aneurysm measuring 5.5 mm x 3 mm x 3 mm with a wide neck (Figure 3).

**Figure 4:**
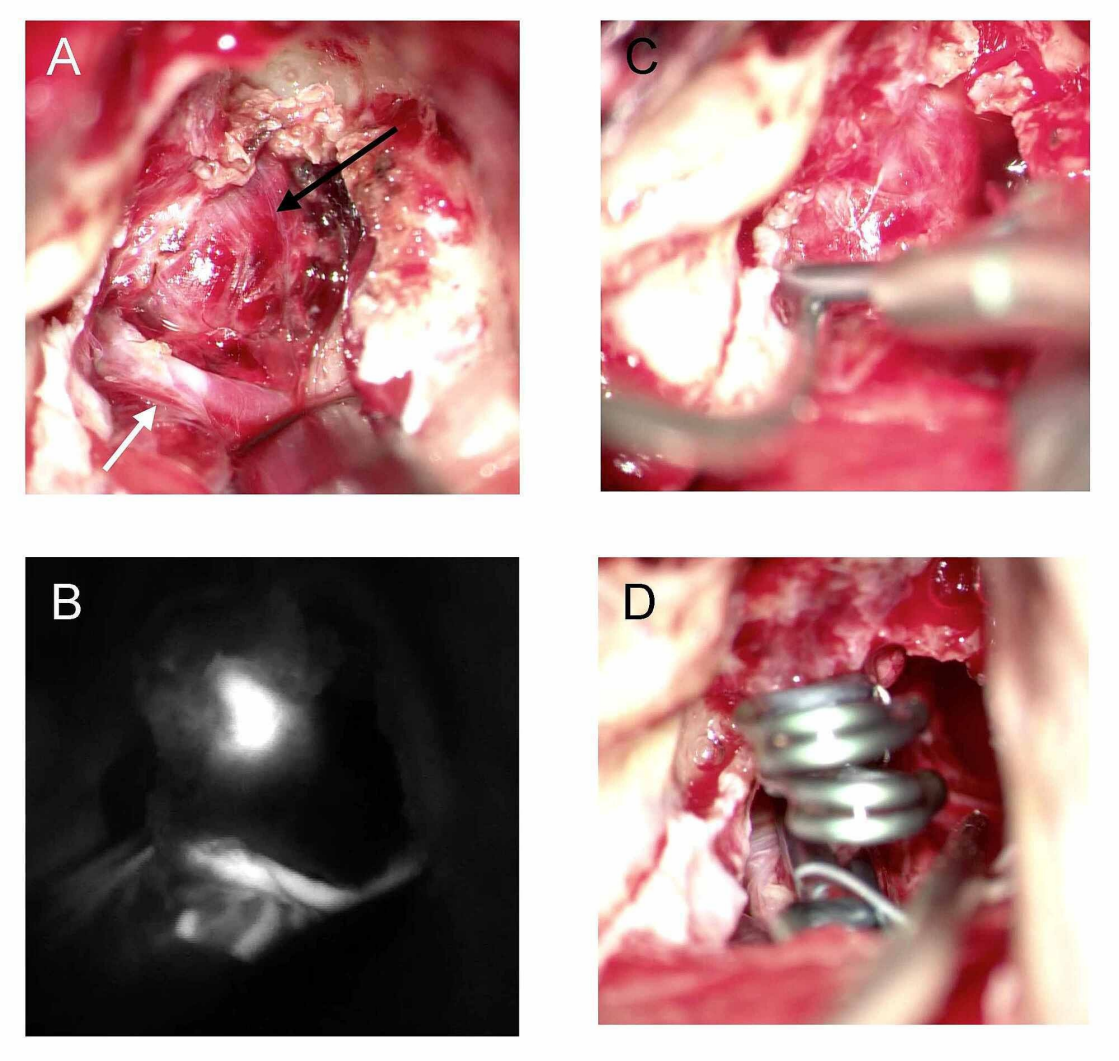
Intraoperative imaging Left translabyrinthine approach to the distal AICA aneurysm. (A) Aneurysm (black arrow) and feeding artery (white arrow). (B) Intraoperative indocyanine green administration shows the feeding artery and aneurysm. (C, D) A total of three clips were placed across the entire aneurysm neck and dome in a “fence post” fashion for complete occlusion. AICA, Anterior inferior cerebellar artery.

On the second hospital day, the patient underwent clip ligation of this aneurysm via a TL approach (Figure 4). The details of that approach are well known [12]. Briefly, the patient was positioned with his head turned 60 degrees to the right. A standard mastoidectomy, skeletonization of the sigmoid sinus, and removal of the bony labyrinth gave access to the IAC. The bone over the IAC was carefully removed with a diamond burr and microsurgical instruments. Dedicated facial nerve monitoring and stimulation were used throughout the procedure (NIM 2.0, Medtronic, Minneapolis, MN, USA). The aneurysm was surrounded by dura and “plugged” into the opening of the IAC. Due to the large amount of subarachnoid hemorrhage, careful evacuation of blood products was necessary to identify the proximal and distal AICA. Given the wide neck of the aneurysm and the small parent artery, three aneurysm clips were placed across the entire aneurysm in a “fence post” fashion to occlude both the aneurysm and the parent artery (Figure 2). At surgery, the dome of the aneurysm was partially embedded in the IAC and appeared larger than that on angiography (approximately 10 mm x 5 mm x 5 mm), indicating that it had partially thrombosed. The large volume of the subarachnoid blood and thickened arachnoid made identification of the facial nerve difficult except in the IAC, where it was easily stimulated. The facial nerve could also be stimulated at the brainstem with high current (1 mA).

Follow-up DSA revealed total occlusion of the aneurysm with patent proximal AICA and distal AICA filling retrograde from collaterals (Figure 3). During his ICU stay, the patient was extubated, weaned from ventricular drainage, and returned to normal consciousness and full strength except for persistent left facial weakness. He was subsequently discharged to a rehabilitation center and recovered well with a modified Rankin Scale of 1. His left facial nerve paralysis persisted at one-year follow-up (House-Brackmann V).

## Discussion

AICA anatomy and aneurysm location

The most widely used anatomical classification of AICA divides it into four segments: (1) anterior pontine segment (A1) from AICA origin to olivary prominence, (2) lateral pontine segment (A2) between olivary prominence and flocculus, (3) flocculopeduncular segment (A3) between the flocculus and cerebellopontine fissure, and (4) cortical segment (A4) [13]. Typically, two branches of AICA emerge near facial-vestibulocochlear nerve complex to form rostal/lateral and caudal/medial trunks. The lateral A2 segment is further divided into premeatal segment anterior to IAC, meatal segment at the IAC, and postmeatal segment posterior to IAC. The meatal segment often gives crucial branches including the internal auditory artery (IAA, also called labyrinthine artery) that supplies CNs VII and VIII, recurrent perforating arteries, and the subarcuate artery [3,13,14]. AICA aneurysms can either originate proximally from the origin of the AICA at the BA-AICA junction (A1) or distally from AICA itself or its branches (A2-A4), with the former more common than the latter [2,4]. As for distal AICA aneurysms, Mizushima reported that most distal AICA aneurysms (94%) originate from lateral branch, close to IAC [3]. Unruptured AICA aneurysm close to meatal segment can be misdiagnosed as VS [15,16]. Yamakawa et al. classified these aneurysms into three types with respect to the IAC: (1) outside of IAC (remote), (2) partially in the IAC (plugged), and (3) entirely in the IAC (buried) [6]. The aneurysm in this report was a type 2 (plugged). 

Clinical presentations

Acute SAH is the most common presentation in patients with AICA aneurysms [2,6,17]. Apart from typical “worst headache of my life,” nausea, and altered mental status, patients often also suffer from ipsilateral hearing loss, otalgia, dizziness, and vertigo secondary to cranial nerve VIII involvement, and occasionally other cranial neuropathies [9]. Yamakawa et al. summarized the clinical presentation of numerous patients with distal AICA aneurysms and hearing loss was common [6].

Diagnosis

The diagnosis of AICA aneurysm is usually straightforward with DSA or CTA. Some patients who present with headache, hearing loss, or other neurological symptoms may undergo MRI first. Differentiating a VS from an aneurysm can be difficult based on MRI alone. Marchini et al. proposed two features on MR imaging to distinguish AICA aneurysms from VS: absence of IAC enlargement and the “blurry dot sign” [16]. The sign consists of hyperintensity on pre-contrast and hypointensity on post-contrast T1-weighted imaging from blood flow artifacts within the aneurysm sac [16]. Our patient’s initial MRIs showed a stable homogeneously enhancing mass which enlarged and showed an area of signal loss on the post-contrast T1-weighted image six days prior to rupture, likely secondary to the disruption of intra-aneurysmal blood flow and thrombus formation. 

Surgical management

The most commonly reported surgical intervention for occlusion of an AICA aneurysm is clip ligation via a retrosigmoid (RS) craniotomy. In the literature, there are only two cases of AICA aneurysm which were approached via a TL corridor. Gonzalez et al. used this approach for clip ligation of a proximal AICA aneurysm that was causing brainstem compression [2]. Diaz et al. reported a Yamakawa type 3 labyrinthine artery aneurysm, which was originally suspected to be a VS [15]. The patient presented with right hearing loss, facial paralysis, and hypoesthesia. Our patient is the third reported AICA aneurysm case, which underwent a TL approach for aneurysm clipping. We utilized this approach based on the aneurysm location (Yamakawa type 2) and the patient’s prior hearing loss. The majority of the surgical experience with the TL approach is from the management of VS, and this exposure provides early identification of the facial nerve. In our case, facial nerve function was already impaired by the aneurysm and subsequent rupture. Although the proximity of the aneurysm to the IAC might make this approach seem risky, the position of the aneurysm on the distal AICA makes the TL approach ideal for early identification of the aneurysm. With many distal AICA aneurysms, the entire aneurysm will need to be occluded along with the parent artery because the distal vessel is small, and the aneurysm includes the majority of the circumference of the distal vessel. The TL approach facilitates bringing aneurysm clips to this location. Unruptured aneurysms, on the other hand, might be more easily approached via the familiar RS approach where visualization is not affected by copious blood products. Even in this situation, a TL approach will undoubtedly bring the distal AICA aneurysm into view faster with less cerebellar retraction.

Endovascular techniques

Multiple methods have been applied endovascularly for AICA aneurysm embolization, such as coils, n-buytlcyanoacrylate, ethylene vinyl copolymer, and flow diverters [4,7,8,18,19]. In general, endovascular management is appropriate for AICA aneurysms at the AICA-BA junction. Distal AICA aneurysms, on the other hand, may be impossible to directly catheterize, and occlusion of AICA proximal to the aneurysm to interrupt blood flow to the aneurysm may be the only option. However, proximal parent artery occlusion (PAO) carries the risk of brainstem/cerebellar infarction [1]. Some hypothesize that occlusion of AICA distal to IAA is safe with minimal consequences [11]. Several cases of PAO with minimal subsequent neurological deficits have been reported [8]. Hou et al. found that in 16 A1-A2 segment AICA aneurysms endovascularly treated with PAO, 11 recovered without neurologic deficits [14]. Incomplete endovascular treatment carries associated risks such as aneurysm enlargement and re-rupture [7]. Endovascular treatment of such distal aneurysms, therefore, carries risks of incomplete treatment, parent artery perforation, infarction, and inability to access the distal AICA vessel.

Outcome

Patients with AICA aneurysms often achieve a good clinical outcome even with aneurysm rupture [2,4,10,13]. Lv et al. reviewed a total of 47 AICA aneurysms treated either surgically or endovascularly and found similar outcomes with both [9]. We summarize case series with five or more AICA aneurysms that have reported the outcome in Table 1 [2,4,10,17,20]. In the table, we combined excellent and good outcomes together. In summary, on an average, 73/83 (88.0%) patients had a favorable outcome. 

**Table 1:** Outcome of AICA aneurysms AICA, Anterior inferior cerebellar artery.

Study	Total patients	Aneurysm ruptured	Patients with follow-up	Good outcome N/%	Mean interval (months)
Li et al., 2012 [17]	6	5	6	6/100%	68
Tokimura et al., 2012 [10]	9	8	9	7/77.8%	N/A
Suh et al., 2011 [4]	8	5	8	8/100%	39.9
Gonzalez et al., 2004 [2]	34	21	19	16/84.2%	41
Drake et al., 1996 [20]	41	35	41	36/87.8%	N/A

## Conclusions

Distal AICA aneurysms are rare, can present with hearing loss and facial palsy, and have a similar appearance to VS on MRI. Once recognized, endovascular or open surgical clip ligation can be used to secure the aneurysm. For distal AICA aneurysms in cases without useful hearing, clip ligation via a TL approach is an effective option.
